# Supplementary material for: Maximally informative feature selection using Information Imbalance: Application to COVID-19 severity prediction
Source: Sci Rep. 2024 May 10;14:10744. doi: 10.1038/s41598-024-61334-6 (PMC11087653; doi:10.1038/s41598-024-61334-6)
Supplement: Supplementary file 1 — Supplementary Information. [file 41598_2024_61334_MOESM1_ESM.pdf]

Supplementary Information
Maximally informative feature selection using Information Imbalance: Application to COVID-19 severity prediction

Romina Wild[1], Emanuela Sozio[2,3], Riccardo G. Margiotta[1], Fabiana Dellai[2], Angela Acquasanta[2], Fabio Del Ben[4], Carlo Tascini[2,4], Francesco Curcio[2,3], and Alessandro Laio*[1,5]

[1]International School for Advanced Studies (SISSA), Via Bonomea 265, Trieste, Italy
[2]Infectious Disease Unit, Azienda Sanitaria Universitaria Integrata di Udine (ASU FC), Via Pozzuolo 330, Udine, Italy
[3]University of Udine, 33100, Via delle Scienze 206, Udine, Italy
[4]Department of Medicine (DAME), University of Udine, Via Palladio 8, 33100, Udine, Italy
[5]The Abdus Salam International Centre for Theoretical Physics (ICTP), Strada Costiera 11, Trieste, Italy
*laio@sissa.it

February 2024

1 Optimal feature sets of different sizes

The globally best n-plets of features as a function of n (until n=13), corresponding to Fig. 2a of the main paper:

1. BNP
2. AT3, IP10
3. TC, A-a gradient, IP10
4. FLNEU%, AT3, IP10, ASMA
5. FLNEU%, AT3, IP10, ACARG, ASMA
6. FLLINF%, AT3, IL-10, TROP, ANCA1, ASMA
7. GOT, FLNEU%, AT3, IL-10, TROP, ANCA1, ASMA
8. Steroid therapy, FLNEU%, GPT, AT3, IL-10, TROP, ANCA1, ASMA
9. Hepatopathy, FLLINF%, GPT, AT3, IL-10, TROP, ANA1, ANCA1, ASMA
10. Hepatopathy, FLLINF%, GPT, AT3, IL-10, ACARG, TROP, ANA1, ANCA1, ASMA
11. Hepatopathy, steroid therapy, potassium sparing diuretics, FLLINF%, GOT, AT3, IL-10, TROP, ANA1, ANCA1, ASMA
12. Hepatopathy, steroid therapy, potassium sparing diuretics, FLEOS%, FLNEU%, GPT, AT3, IL-10, TROP, ANA1, ANCA1, ASMA
13. Hepatopathy, steroid therapy, potassium sparing diuretics, FLLINF, FLEOS, FLNEU%, GPT, AT3, IL-10, TROP, ANA1, ANCA1, ASMA

2 Predictive power for patients without the optimal input tuples

The optimal 13-plet from our study is only available for about 100 patients. Our method, however, can find for each patient their patient-specific best n-plet, with a slight loss of predictive accuracy when averaged over all patients (Fig. S1). This makes sense since 102 patients have the features of the best 13-plet, while the rest the patients do not have complete data for these features. Hence, their respective Δ_w-optimized n-tuple has higher (worse) Information Imbalance than the optimal 13-plet, which influences the prediction accuracy.

The patient-specific optimal n-plets were found in a leave-one-out (LOO) approach by considering all features that were present in the respective patient, then beam-searching over these starting from the 1-plets. For each of these feature tuples the Weighted Information Imbalance is calculated using all the patients who have full information in these features, and the search is stopped when the Information Imbalance flattens or starts increasing. In this way, the patient-specific optimal tuple is found, and along with it the optimal dimensionality. Then we performed a 10-NN prediction of severity for each patient, using their optimal n-plet of features in a LOO cross validation, where we use all other patients who share the same features as training set. As default, the algorithm only considers possible feature tuples which are available in at least 100 patients and have a base-2 Jensen-Shannon divergence of ≤ 0.06, in order to be representative of all classes in the full set.

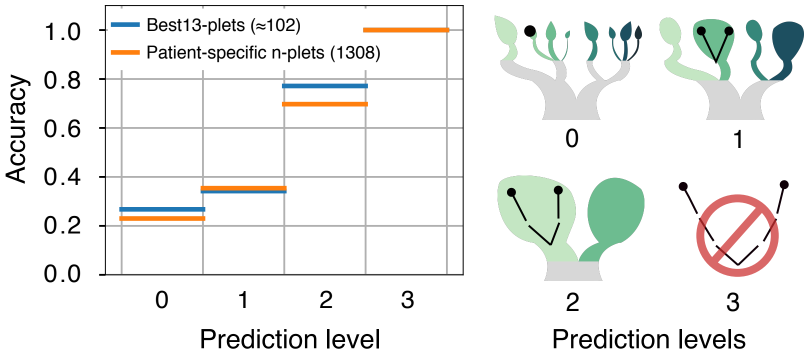

Figure S1. Accuracies of 10-NN predictions at given prediction levels by the optimal 13-plets (averaged over the ten best), which are available in roughly 102 patients, *vs.* accuracies using for each patient in the database (1308 patients) their optimal input feature tuple. The accuracy corresponds to the fraction of patients predicted correctly at a given prediction level. The prediction level corresponds to the maximum tolerated distance of the true *vs.* the predicted class on the severity tree, as depicted on the right. Together, the lines of one color can be considered the CDF of the fraction of patients predicted correctly.

3 Accuracy of prediction compared to regularized classifiers

Information Imbalance is not a classification method. However, in the application described in this work the ground truth metric is defined on a severity tree. Its leaves can be considered as categories (classes) which can be used as a target for a classification method. The approach in the main paper introduces class-corrected Weighted Information Imbalance as a novel filter method to do feature selection, which is followed by classification. Here, a comparison with regularized classifiers, which do not employ explicit prior feature selection, is presented. The sklearn [6] implementations of

1. L_1 (lasso) regularized logistic regression classification
   sklearn.linear_model.LogisticRegression with
   penalty='l1', C=1 or 0.04, max_iter=200, class_weight='balanced', solver='liblinear', tol=0.01

2. Regularized sparse SVC
   sklearn.svm.SVC with C=10 or 1, kernel='rbf', gamma='auto', class_weight='balanced'

were tested. The results are presented in Fig. S2 and build on Fig. 3 of the main paper. Indeed, in terms of general prediction accuracy these regularized classification methods perform at least as good, sometimes better, as the filter models. When considering minority classes only (leaving out the biggest

class of patients who did not have a complication or event), then Information Imbalance followed by SVC classification outperforms all other models, especially for the most exact prediction level (0). For all prediction levels, modestly regularized ($C = 1$) sparse SVC yields the second best results.

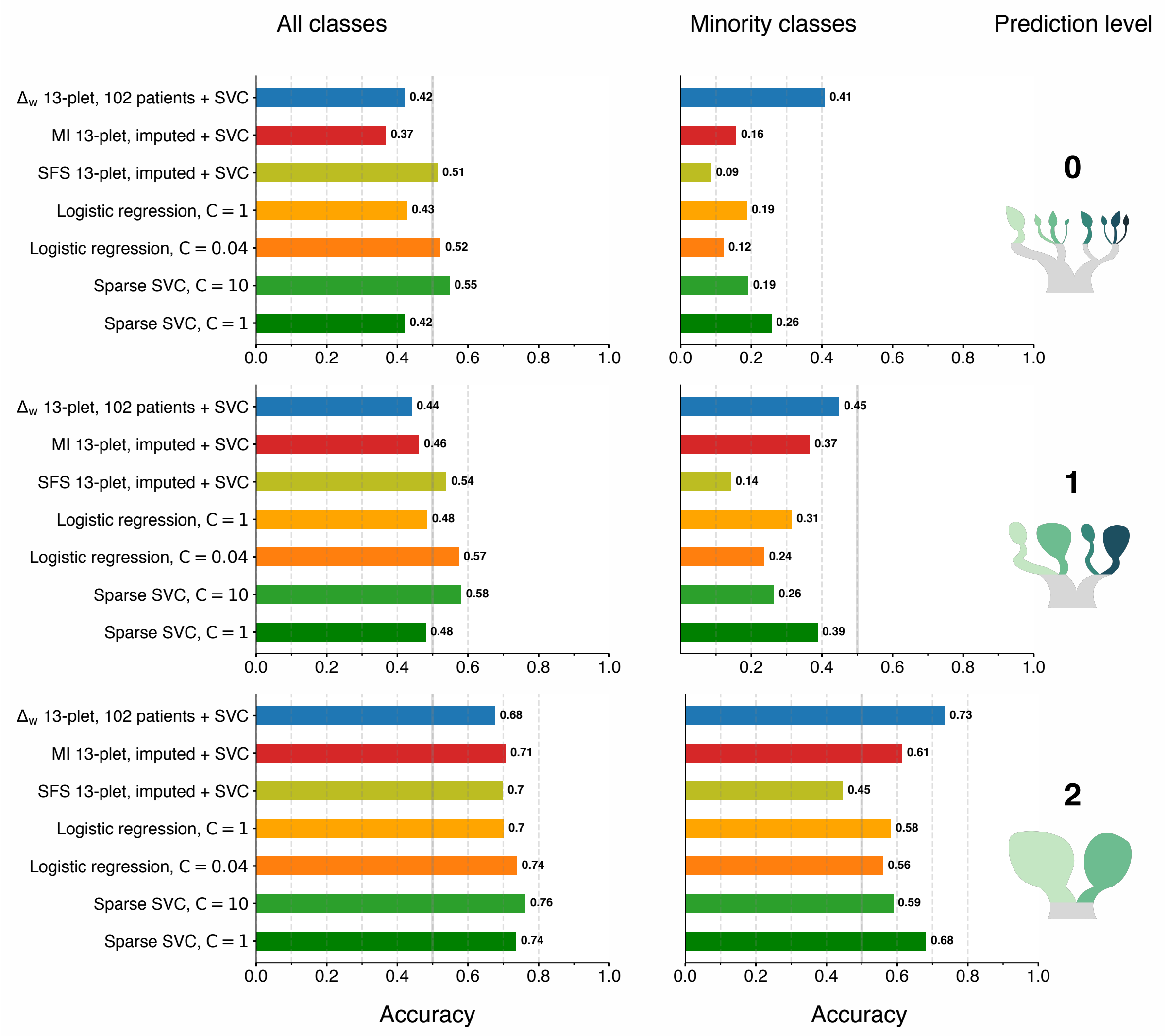

Figure S2. Accuraciess of prediction of several models, corresponding to Fig. 3 of the main paper (SVC only), and compared with two classifiers, logistic lasso regression and sparse SVC. The accuracy corresponds to the fraction of patients predicted correctly at a given prediction level. The prediction level corresponds to the maximum tolerated distance of the true *vs.* the predicted class on the severity tree, as depicted on the right. 0: exact class predicted; 1: event and complication predicted; 2: event predicted.

4 The performance of other approaches using feature tuples of various sizes

The algorithm described in this work provides an automatic way to determine the optimal size of the predictive tuple at the feature selection stage - an advantage that other filter methods do not have. In the main paper we compare WII selected 13-plets with 13-plets selected with two other methods, Mutual Information (MI) and sequential forward selection (SFS). Since these two methods do not provide a determination of optimal tuple size at the feature selection stage, the comparison can be performed only at the prediction accuracy stage. The results of this analysis are shown in Fig. S3. The performance of these approaches is almost independent on the tuple size, for sizes between 10 and 15.

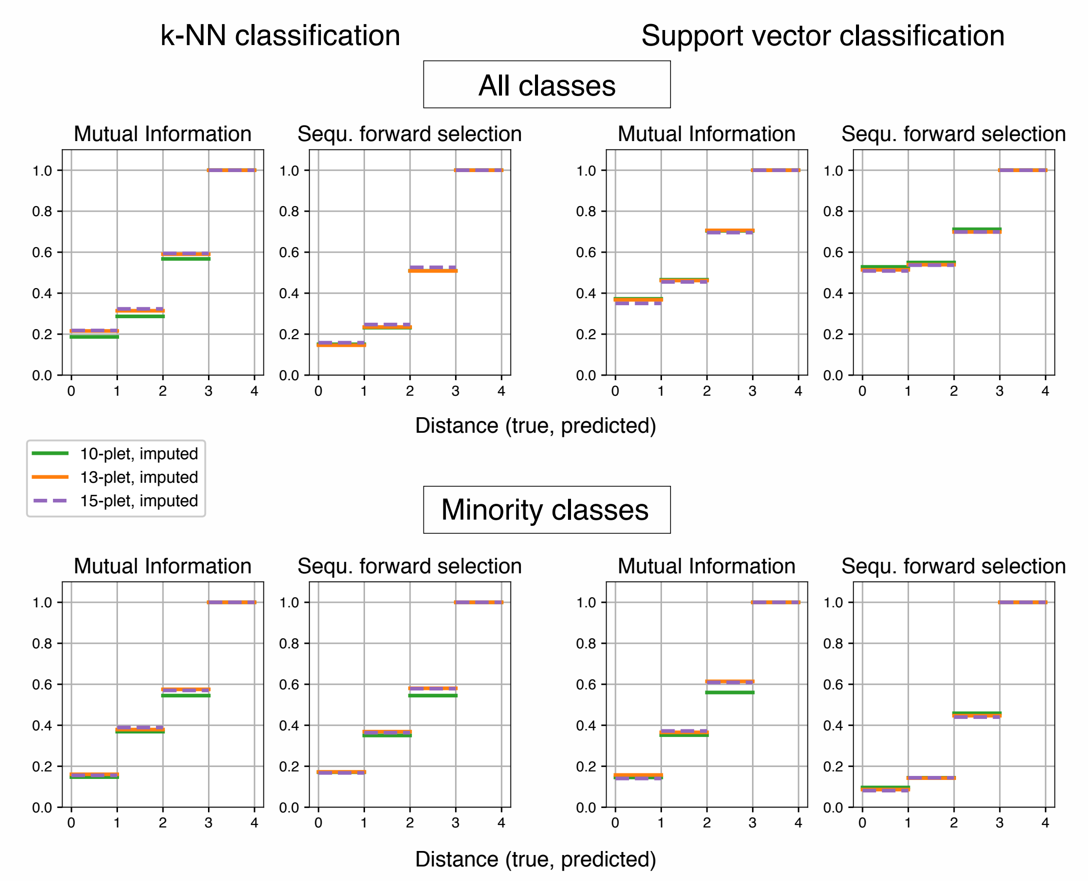

Figure S3. Accuraciess of k-NN and SVC predictions using optimized tuples of different sizes. The tuples were optimized by feature selection with Mutual Information (MI) and sequential forward selection (SFS). The accuracy is described in Fig.S2.

5 Correlation and Information Imbalance between input features

5.1 Information Imbalance and correlation within Δ_w-optimized n-plets

The Weighted Information Imbalance approach (main paper) automatically leads to the selection of tuples of features which are practically uncorrelated. This is demonstrated by calculation of the Pearson correlation coefficient and the pairwise classic Information Imbalance (introduced in ref. [3]) for all the numerical features contained in the Δ_w-optimized nplets (Fig. S4.). The mean of pairwise correlations of numerical features in *e.g.* the best 13plet (Fig. S4 blue outline), is $r = 0.02$, and the pairwise Information Imbalances mean is $\Delta = 0.96$, both of which point towards a high degree of orthogonality.

5.2 Information Imbalance between numerical patient features

We also use the classic Information Imbalance to investigate the relationships between the input features. We consider the 90 numerical input features for which it is possible to estimate the standard Information Imbalance introduced in ref. [3]. We computed the Information Imbalance Δ between each pair of features using the implementation in the Python package DADApy [2]. $\Delta(A \to B)$ is close to zero if feature A predicts feature B well. It is close to one if feature A does not provide information on feature B. For each pair of features we also computed the standard Pearson and Spearman correlation coefficients r and ρ, which are ± 1 in the case of a perfect positive or negative correlation, and 0 if there is no correlation. If two features correlate strongly, $\Delta(A \to B)$ and $\Delta(B \to A)$ should both be small and similar numbers, if both predict each other to an equal amount. However, if one feature predicts the other, but not vice versa, there exists an asymmetric correlation, and this is reflected in an asymmetric Information Imbalance. This phenomenon, as we will see, is not captured by Pearson and Spearman correlations.

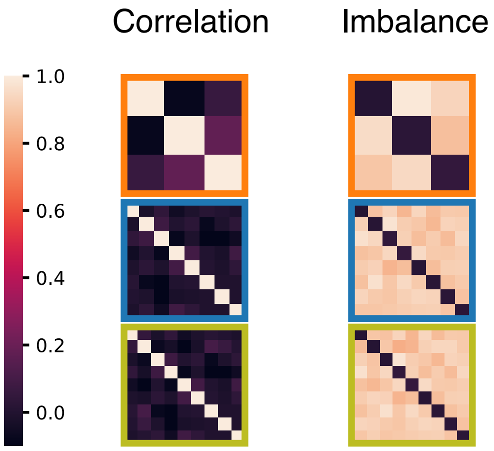

Figure S4. The pairwise Pearson correlation heat mat of the numerical features of the Δ_w-optimized n-plets(3, 13, and 20) from Figure 2.a of the main paper, and the pairwise, classical Information Imbalances of the same n-plets.

In the table in Fig. S5a, we report the Information Imbalance and the correlation coefficients between the 20 pairs of features with the lowest $\Delta(A \to B)$. To highlight some possible relationships, we plot some of these feature as a function of each other in the bottom panels (Fig. S5b).

The Information Imbalance faithfully captures features which have strong correlations with each other. The top-eight positive correlation couples (all $r > 0.8$) are contained in the top-20 Information Imbalances. A clear sanity check is displayed in the first rows: the two different laboratory methods for the glomerular filtration rate (GFR and GFR.1), which hold the same values, display perfect correlations and extremely low Information Imbalances. This is also true for the prothrombin time (- and international normalized) ratios (PT/INR and PTR), where one is just a normalized version of the other. Correlation and Information Imbalance pick up on the linear relationship between hematocrit (EHCT) and hemoglobin (EHB). EHCT is the percentage of volume occupied by red blood cells relative to whole blood, and therefore is often related to hemoglobin (EHB). Also, the strongest negative correlation pairing is in the top-20 imbalance table (row 6). Thus the strongest correlations account for nine rows in the top-20 Information Imbalances. The other eleven rows are made up by pairings which have less strong correlations, but six of them have high asymmetries in their Information Imbalances towards each other (yellow rows in S5a), describing a relationship where one variable is more informative about the other than *vice versa*. These six pairings have predominantly very low correlations, showcasing that correlation fails to identify these asymmetric relationships. The effect is especially pronounced in row five, where PaO2/FiO2 (oxygen partial pressure over fractional inspired oxygen) has a low Information Imbalance towards PaO2 (oxygen partial pressure) and such explains this feature space well, while the same is not true *v.v.* This can be used as a proof of concept because indeed PaO2/FiO2 is the value of PaO2 divided by FiO2 (fractional inspired oxygen) - a simple relationship via one confounding variable which is not detected by correlation (r=0.177). It should be noted that, from a clinical point of view, measuring the PaO2/FiO2 ratio can become very challenging: if patients are not on invasive mechanical ventilation, it is almost impossible to know the exact FiO2, because the devices deliver a variable inspired oxygen concentration. Information Imbalance also detected similar cases where the exact relationship is not known: Here we report asymmetric relationships between troponin (TROP), a well-known marker of cardiac injury, and tissue damage marker fibrinogen (FIBCL), as well as between the immunoglobulins IGM / IGG and the high-density lipoprotein (HDL). TROP values are more predictive of FIBCL values than the other way around. Fibrinogen is a plasma acute-phase reactant protein produced by the liver and is a major coagulation factor. Its concentration increases with inflammation, and it is traditionally considered a risk factor for cardiovascular disease [8, 5], which might explain the connection to troponin. Troponin, on the other hand, is a very specific marker: recent studies showed that troponin dosage should be considered as a prognostic indicator in all patients with moderate/severe COVID-19 at hospital admission and in the case of clinical deterioration [**Lippi2020**]. Retrospective data have placed a strong emphasis on the possibility that acute myocardial injury represents a critical component in the development of serious complications in patients hospitalized with COVID-19 [1, 7, 4]. To the best of our knowledge, there is no literature concerning the exact relationships between IGM / IGG and HDL.

a

No.	Feature A	Feature B	Pearson r(A, B)	Spearman ρ(A, B)	Δ(A→ B)	Δ(B → A)
1	GFR.1	GFR	1.000	1.000	0.037	0.037
2	PT/INR	PTR	1.000	0.998	0.049	0.050
3	EHCT	EHB	0.981	0.976	0.236	0.261
4	EWBC	FLNEU	0.874	0.959	0.276	0.283
5	PaO2/FiO2	PaO2	0.177	0.279	0.351	0.805
6	FiO2	PaO2/FiO2	-0.906	-0.770	0.425	0.441
7	EMCH	EMCV	0.947	0.916	0.448	0.450
8	EHCT	ERBC	0.861	0.853	0.527	0.541
9	CRE	GFR	-0.533	-0.842	0.560	0.592
10	CRE	GFR.1	-0.533	-0.842	0.560	0.579
11	EHB	ERBC	0.820	0.819	0.561	0.592
12	Oxygen saturation	PaO2	0.599	0.807	0.571	0.593
13	BILD	BILT	0.962	0.874	0.573	0.598
14	TROP	FIBCL	-0.496	-0.507	0.574	0.713
15	IGM	HDL	-0.003	0.109	0.612	0.810
16	IGG	HDL	-0.295	-0.055	0.628	0.876
17	A-a gradient	PaCO2	-0.455	-0.695	0.651	0.691
18	BNP	Birth	-0.504	-0.708	0.662	0.792
19	TROP	Anion gap	0.063	-0.382	0.699	0.858
20	HBA1CM	IGA	0.100	0.042	0.711	0.804
...	...	...	...	...	...	...
21	BMI	GFR	-0.077	-0.044	1.029	1.040

b

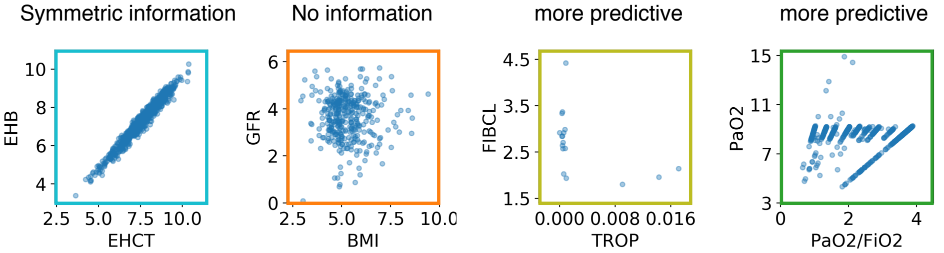

Figure S5. **a**: Features ordered according to the lowest Information Imbalances towards another feature, and their Pearson and Spearman correlation coefficients. Yellow colored rows have notably asymmetric Information Imbalances, where $|\Delta(A \to B) - \Delta(B \to A)| > 0.1$. **b**: Scatter plots of several of the feature *vs.* each other from A. The values are the normalized features.

While causality-based feature selection methods only aim at finding causalities between the predicting features and the target classes [9], we point out here that inter-feature asymmetric relationships, such as inter-feature causalities, could also be important. They are not captured by standard correlation analyses, and they could lead to identify redundancy effects when tuples of features are used for predictive purposes. This pairwise Information Imbalance between the features was used as a sanity check of the prediction tuples used in the previous section. All Δ_w-optimized tuples were indeed non-redundant, with Information Imbalances of > 0.85 between each other.

6 The stability of selected features the with small random numbers in the input

Since the addition of the small random numbers to degenerate inputs renders Δ_w a stochastic variable, we probed the influence of changing the random seed in ten instances. The Information Imbalances of optimal tuples with the same tuple sizes are mostly identical, up to the second digit, with standard deviations on the order of 10^{-4}. Also the optimal tuples themselves showed little variability, as seen in Fig. S6.

References

[1] Tao Chen et al. “Clinical characteristics of 113 deceased patients with coronavirus disease 2019: retrospective study”. In: *BMJ* 368 (2020). DOI: https://doi.org/10.1136/bmj.m1091. URL: https://www.bmj.com/content/368/bmj.m1091.

[2] Aldo Glielmo et al. “DADApy: Distance-based analysis of data-manifolds in Python”. In: *Patterns* 3.10 (2022), p. 100589. ISSN: 2666-3899. DOI: https://doi.org/10.1016/j.patter.2022.100589. URL: https://www.sciencedirect.com/science/article/pii/S2666389922002070.

[3] Aldo Glielmo et al. “Ranking the information content of distance measures”. In: *PNAS Nexus* 1.2 (Apr. 2022). ISSN: 2752-6542. DOI: https://doi.org/10.1093/pnasnexus/pgac039. URL: https://doi.org/10.1093/pnasnexus/pgac039.

[4] Tao Guo et al. “Cardiovascular Implications of Fatal Outcomes of Patients With Coronavirus Disease 2019 (COVID-19)”. In: *JAMA Cardiology* 5.7 (July 2020), pp. 811–818. ISSN: 2380-6583. DOI: https://doi.org/10.1001/jamacardio.2020.1017. URL: https://doi.org/10.1001/jamacardio.2020.1017.

[5] Gordon DO Lowe, Ann Rumley, and Ian J Mackie. “Plasma fibrinogen”. In: *Annals of Clinical Biochemistry* 41.6 (2004), pp. 430–440. DOI: https://doi.org/10.1258/0004563042466884. URL: https://doi.org/10.1258/0004563042466884.

[6] F. Pedregosa et al. “Scikit-learn: Machine Learning in Python”. In: *Journal of Machine Learning Research* 12 (2011), pp. 2825–2830.

[7] Shaobo Shi et al. “Association of Cardiac Injury With Mortality in Hospitalized Patients With COVID-19 in Wuhan, China”. In: *JAMA Cardiology* 5.7 (July 2020), pp. 802–810. ISSN: 2380-6583. DOI: https://doi.org/10.1001/jamacardio.2020.0950. URL: https://doi.org/10.1001/jamacardio.2020.0950.

[8] Thomas M Stulnig. “C-reactive protein, fibrinogen, and cardiovascular risk”. In: *New England Journal of Medicine* 368.1 (2013), pp. 84–86. DOI: https://doi.org/10.1056/NEJMc1213688.

[9] Kui Yu et al. “Causality-Based Feature Selection: Methods and Evaluations”. In: *ACM Comput. Surv.* 53.5 (Sept. 2020). ISSN: 0360-0300. DOI: https://doi.org/10.1145/3409382.

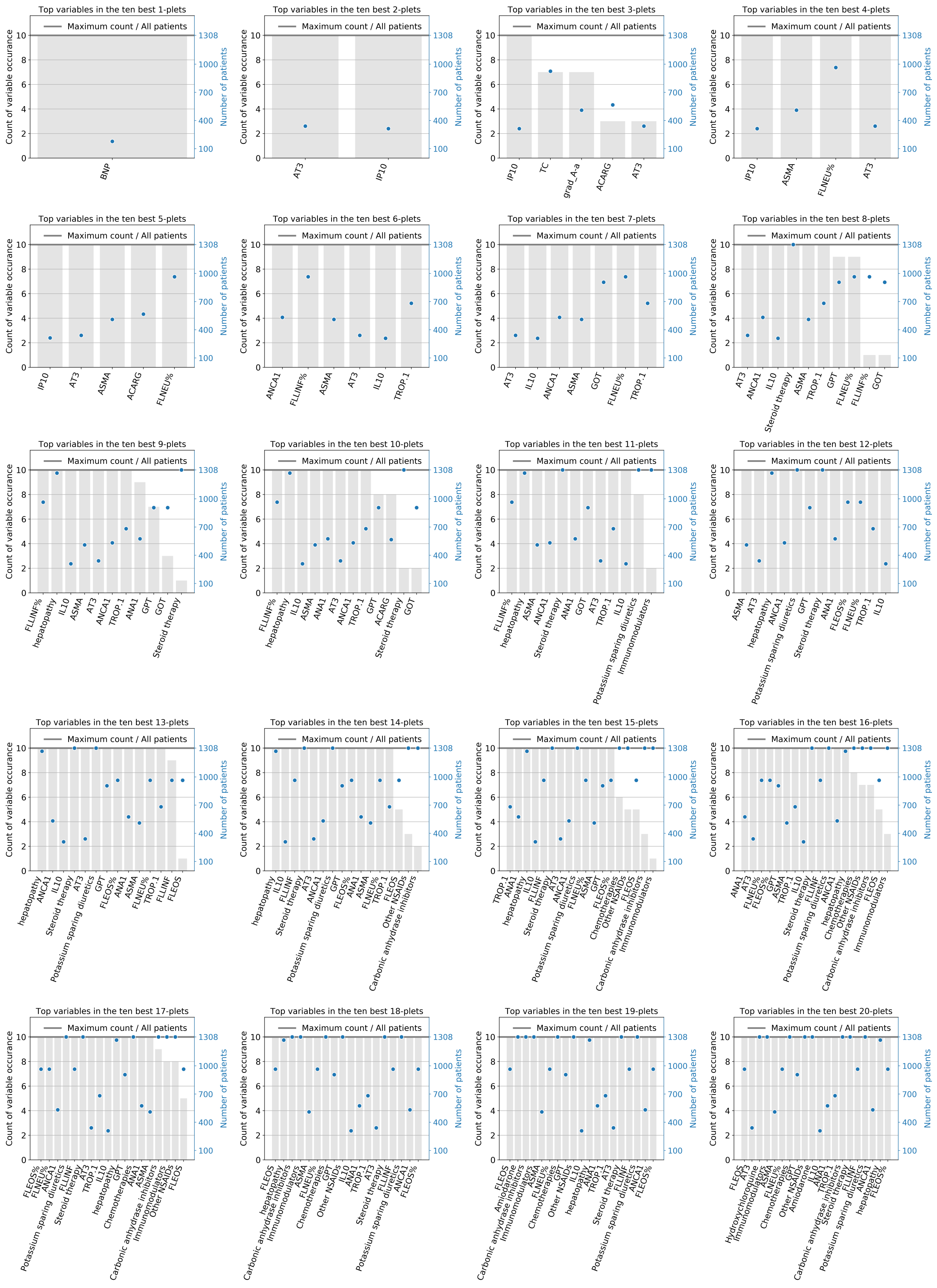

Figure S6. The panels show the variables chosen in each of the Δ_w feature tuples of different size across ten implementations with different small random numbers added to the degenerate input features. The grey bars corresponding to the left axis shows in how many of the 10 copies a feature was chosen, the blue dots and right axis indicate the number of patients for whom the feature was available.
